# Exo-Erythrocytic Development of Avian Haemosporidian Parasites in European Owls

**DOI:** 10.3390/ani12172212

**Published:** 2022-08-28

**Authors:** Mikas Ilgūnas, Tanja Himmel, Josef Harl, Mindaugas Dagys, Gediminas Valkiūnas, Herbert Weissenböck

**Affiliations:** 1 Nature Research Centre, Akademijos 2, 08412 Vilnius, Lithuania; 2 Department for Pathobiology, Institute of Pathology, University of Veterinary Medicine Vienna, Veterinaerplatz 1, 1210 Vienna, Austria

**Keywords:** haemosporidian parasites, birds, exo-erythrocytic development, tissue stages, Strigiformes

## Abstract

**Simple Summary:**

Avian haemosporidians of the genera *Plasmodium*, *Haemoproteus,* and *Leucocytozoon* are vector-borne blood parasites, which commonly infect birds all over the world, except for Antarctica. Although called blood parasites, these pathogens develop not only in the blood cells of vertebrate hosts, but also in the tissues of various organs. While the blood stages have been studied quite intensively, the tissue stages, patterns of their development, and their effect on the vertebrate host are not well understood, especially in wild, non-passerine birds. The present study aimed at gaining new knowledge about avian haemosporidian parasites naturally infecting owls in Austria and Lithuania. Organ samples of 121 owls were investigated for blood parasites using molecular and histological methods. Over 70% of the owls were infected, revealing seven new genetic variants (lineages) of avian haemosporidian parasites. Tissue stages of *Leucocytozoon* spp. and *Haemoproteus syrnii*, a common parasite in owls, were discovered, providing new insights into the parasites’ tissue development. This study contributes new knowledge to a better understanding of the biodiversity and life cycles of avian haemosporidian parasites. These data are crucial for avian medicine and bird protection and indicate directions for further research on the tissue development of haemosporidian infections.

**Abstract:**

Avian haemosporidian parasites (Haemosporida, Apicomplexa) are globally distributed and infect birds of many orders. These pathogens have been much investigated in domestic and wild passeriform birds, in which they are relatively easy to access. In birds belonging to other orders, including owls (order Strigiformes), these parasites have been studied fragmentarily. Particularly little is known about the exo-erythrocytic development of avian haemosporidians. The goal of this study was to gain new knowledge about the parasites infecting owls in Europe and investigate their exo-erythrocytic stages. Tissue samples of 121 deceased owls were collected in Austria and Lithuania, and examined using polymerase chain reactions (PCR), histology, and chromogenic in situ hybridization (CISH). PCR-based diagnostics showed a total prevalence of 73.6%, revealing two previously unreported *Haemoproteus* and five novel *Leucocytozoon* lineages. By CISH and histology, meronts of several *Leucocytozoon* lineages (lASOT06, lSTAL5, lSTAL7) were discovered in the brains, heart muscles, and kidneys of infected birds. Further, megalomeronts of *Haemoproteus syrnii* (lineage hSTAL2) were discovered. This study contributes new knowledge to a better understanding of the biodiversity of avian haemosporidian parasites infecting owls in Europe, provides information on tissue stages of the parasites, and calls for further research of these under-investigated pathogens relevant to bird health.

## 1. Introduction

Avian haemosporidian parasites (Haemosporida, Apicomplexa) are widespread pathogens infecting birds all over the world, with the exception of Antarctica [1]. Species belonging to the genera *Haemoproteus*, *Leucocytozoon*, and *Plasmodium* are obligate heteroxenous pathogens transmitted by blood-sucking dipterans—louse flies (Hippoboscidae) and biting midges (Ceratopogonidae), black flies (Simulidae), and mosquitoes of various genera (Culicidae), respectively [2]. While being rich in the number of morphologically described species [3,4], an even more remarkable genetic diversity of avian haemosporidian pathogens has been revealed by the introduction of molecular methodologies to this research field [5,6,7].

Over the years, avian haemosporidians have attracted much attention from the scientific community, and many studies have been dedicated to investigations of the taxonomy, genetic diversity, ecology, evolutionary biology, and genomics of the parasites [1,6,7,8,9,10,11]. However, the exo-erythrocytic development of these pathogens remains poorly understood. This is unfortunate because haemosporidians can cause marked damage to the internal organs of their avian hosts and certainly are important for bird health [12]. While the erythrocytic stages of avian haemosporidians are relatively easy to access for research purposes due to their presence in the peripheral blood, this is not the case in exo-erythrocytic stages (or tissue stages). To access these stages, tissue samples need to be obtained from dead birds and analyzed using histological protocols [3]. Exo-erythrocytic stages—meronts and megalomeronts—develop in the tissues within various internal organs of the vertebrate host [3]. Tissue meronts (formed by *Haemoproteus*, *Leucocytozoon,* and *Plasmodium*) are thin-walled structures, commonly below 50 µm in length, and are smaller than megalomeronts (formed by *Haemoproteus* and *Leucocytozoon* spp.). The latter are big structures, frequently exceeding a diameter of 300 µm, which are often roundish or oval in shape and surrounded by a thick capsular-like wall [3,12]. Patterns and sequences of the development of these two types of tissue stages remain insufficiently understood and warrant further research.

Nowadays, investigators are often deterred from studying the exo-erythrocytic development of avian haemosporidian parasites because of the complicated procedures of collecting organ samples from wild birds, complex research methodologies, and bioethical permit-issuing difficulties. In older literature, numerous lethal infections, often attributed to parasites of the genus *Leucocytozoon*, have been documented [12], while *Haemoproteus* parasites, for a long time, were considered relatively benign [13]. The recent introduction of molecular methods facilitating the identification of parasite stages observed in the tissues of their avian hosts allowed for a better understanding of haemosporidian-caused pathologies [14,15,16]. The exo-erythrocytic development and its effect have been studied quite well in several *Leucocytozoon* species using experimental infections [12]. By contrast, in the case of *Haemoproteus* parasites, only the application of methods such as polymerase chain reaction (PCR), chromogenic in situ hybridization (CISH), and single-cell laser microdissection allowed to show that these parasites can also have severe negative effects on their avian hosts [16,17,18,19,20,21,22,23,24,25,26], raising doubts about the previously supposed benignity of these organisms. Further studies using the aforementioned and likely other modern techniques (for example RNAScope technology) are needed to gain a more complete understanding of the haemosporidian exo-erythrocytic development in the avian host and determine its patterns and relation to bird health.

Owls (Strigiformes) are mostly nocturnal birds of prey, especially well adapted for hunting invertebrates and vertebrates, particularly small rodents [27]. More than 200 species belong to this order, many of which are either endangered or near threatened [27]. Numerous studies examined the prevalence of avian haemosporidians in strigiform birds and found that they are readily susceptible to these parasites [28,29,30,31,32,33,34,35,36,37,38,39]. The exo-erythrocytic development has also been fragmentarily investigated in owls, with most of the available information published in a few reports [12,40,41,42,43], and an experimental study [44] from the Americas and Asia; however, from Europe, such studies are absent.

Further investigation of the exo-erythrocytic development of avian haemosporidians in birds belonging to the order Strigiformes is needed. This is particularly true due to the high prevalence of haemosporidians in owls, many species of which are endangered. During the present study, samples from deceased owls were collected in Austria and Lithuania, haemosporidian parasites were identified using DNA barcoding sequences, and their exo-erythrocytic development was investigated by histology and CISH. The main aims of this study were to (i) investigate the parasites infecting the birds belonging to the order Strigiformes, and (ii) gain new information about the exo-erythrocytic development of avian haemosporidian parasites in European owls.

## 2. Materials and Methods

### 2.1. Samples

Owl samples originating from Lithuania (*n* = 37) were collected by the staff of the Kaunas Tadas Ivanauskas Zoological Museum (Kaunas, Lithuania) from 2019 to 2021. All bird individuals were found deceased in the wild, frozen, and brought to the museum for taxidermic purposes. Samples originating from Austria (*n* = 84) were collected during post-mortem examinations of wild owls submitted for routine pathological investigations (*n* = 43) or from injured owls brought to the University of Veterinary Medicine in Vienna (Vetmeduni Vienna) for treatment (*n* = 41) in the period from 2005 to 2018. In total, samples of 121 birds belonging to nine species, *Asio flammeus* (*n* = 1), *Asio otus* (*n* = 30), *Athene noctua* (*n* = 3), *Bubo bubo* (*n* = 10), *Glaucidium passerinum* (*n* = 6), *Strix aluco* (*n* = 39), *Strix uralensis* (*n* = 29), *Strix* sp. (*n* = 1), and *Tyto alba* (*n* = 2) were retrieved from these two institutions.

For molecular diagnostics of haemosporidian infections, pieces of breast muscle, liver, and/or spleen fixed in 96% ethanol (*n* = 80) were used. Additionally, dried blood spots prepared from blood samples obtained prior to the birds’ death (*n* = 41, samples from Austria) were used for extraction of the DNA. The blood samples were obtained during routine blood draws in living birds that were kept for treatment at the service unit for birds and reptiles (Vetmeduni Vienna). For histological examination, tissue samples were collected from internal organs (brain, heart, kidney, liver, lung, spleen, and a piece of pectoral muscle), fixed in 10% neutral buffered formalin, and dehydrated in increasing concentrations of ethanol (70–100%) and isopropanol. After dehydration, tissues were clarified in xylene and embedded in paraffin blocks.

### 2.2. Molecular Diagnostics

#### 2.2.1. DNA Extraction

DNA (desoxyribonucleic acid) of owl samples obtained in Lithuania was extracted applying a protocol designed to extract DNA from skin, hair, and feathers using a lysis buffer (0.1 M Tris, 0.005 M EDTA, 0.2% SDS, 0.2 M NaCl; pH = 8.5) [45]. In short, a small piece of tissue was incubated in 100 µL of lysis buffer with 3 µL proteinase K (Thermo Fisher Scientific, Vilnius, Lithuania) for 3 h at 56 °C. Then the tubes were centrifuged for 10 min at 10,000 RPM, followed by the transfer of the supernatant to a new tube and the addition of absolute EtOH. The tubes were again centrifuged for 10 min at 10,000 RPM and the absolute EtOH was replaced by 70% EtOH. After 5 min of centrifugation at 10,000 RPM, ethanol was removed and the samples were air-dried overnight at room temperature and dissolved in 100 µL 1X TE buffer.

DNA of samples obtained in Austria was isolated using the DNeasy Blood & Tissue Kit (Qiagen, Venlo, Netherlands) following the manufacturer’s instructions with one modification: The DNA was eluted twice with each 100 µL AE buffer, and the second eluate was used as a template for the PCR.

#### 2.2.2. Polymerase Chain Reaction

Samples obtained in Lithuania were screened using PCR protocols amplifying a 478 bp fragment of the cytochrome b gene (*cytB*) (the so-called “MalAvi fragment” [5]) of the parasites. Nested PCR protocols using the primer pairs HaemNFI/HaemNR3 along with HaemF/HaemR2 [46,47] and Plas1F/HaemNR3 with 3760F/HaemJR4 [48] were applied. Conditions for all reactions were maintained as per their original descriptions. In brief, the extracted DNA was diluted to 25 ng/µL and used as a template for the first step of the nested PCR protocols. The product of the first PCR reaction was used as the template for the second step. PCRs were performed in 25 µL reaction volumes containing 12.5 µL of the Dreamtaq Master Mix (Thermo Fisher Scientific, Vilnius, Lithuania), 8.5 µL of nuclease-free water, 1 µL of each primer, and 2 µL of template DNA. A *Haemoproteus* sp.-positive sample and ddH_2_0 as the negative control were used every 14 samples in order to rule out contaminations and evaluate the success of the protocol application. Electrophoresis on a 2% agarose gel using 2 µL of the final PCR product was performed to check for positive amplification.

Samples obtained in Austria were screened using the nested PCR protocol by [47], applying the primer pairs HaemNFI/HaemNR3, HaemF/HaemR2, and HaemFL/HaemR2L. PCRs were performed in 25 µL reaction volumes containing 14.375 µL of nuclease-free water, 5 µL of 5X Green GoTaq Flexi Buffer (Promega, Madison, WI, USA), 2 µL of a MgCl_2_ solution (25 mM), 0.5 µL of the PCR nucleotide mix (10 mM, Promega), 0.125 µL of GoTaq G2 Flexi DNA Polymerase (5 u/µL, Promega), each 1 µL of forward and reverse primers (10 pmol/µL), and 1 µL of the DNA template, or, in case of the second PCRs, 1 µL of the amplicon from the first PCR. The thermal profile of the PCR reactions included an initial 2 min at 94 °C, followed by 35 cycles of 30 s at 94 °C, 30 s at 50 °C, and 1 min at 72 °C, followed by 10 min final extension at 72 °C. For every PCR run, a sample confirmed haemosporidian-positive in previous screenings and nuclease-free water were included as controls. PCR amplification was checked by gel electrophoresis of the 3 µL PCR product on 1% agarose gels stained with Midori Green Advance (Nippon Genetics Europe, Dueren, Germany), and visualization of amplicons using a BioSens SC-Series 710 gel documentation system (GenXpress, Wiener Neudorf, Austria).

#### 2.2.3. Sequencing and Sequence Analysis

Successfully amplified fragments from Lithuanian samples were sequenced using the Big Dye Terminator V3.1 Cycle Sequencing Kit and ABI PRISMTM 3100 capillary sequencing robot (Applied Biosystems, Foster City, CA, USA). The obtained sequences were analyzed using the Geneious Prime 2022.1.1 (Dotmatics, Auckland, New Zealand, https://www.geneious.com, accessed on 12 May 2022) software. PCR amplicons obtained from Austrian samples were sent to Microsynth Austria for bidirectional sequencing. Obtained sequences and electropherograms were analyzed using the software Bioedit (downloaded at https://bioedit.software.informer.com/, accessed on 24 August 2022) [49].

Sequences with ambiguous characters, indicating co-infections, were double-checked and unphased using DnaSP v.6.12.3 (downloaded at http://www.ub.edu/dnasp/, accessed on 24 August 2022) [50]. All sequences were subjected to BLAST search in the MalAvi database (Lund University, Lund, Sweden, http://130.235.244.92/Malavi/, accessed on 12 May 2022) [5] and NCBI GenBank. New lineage names were assigned to haplotypes not matching 100% with previously published *cytB* lineages according to MalAvi rules [5]. Partial *cytB* sequences of all detected haemosporidian parasites were uploaded to GenBank under the accession numbers ON932197–ON932301.

#### 2.2.4. Phylogenetic Analysis

Most of the *Haemoproteus* and *Leucocytozoon* lineages detected in the present study clustered within two major clades containing lineages detected in Strigiformes and birds of other orders. Lineages previously reported from owls and clustering in these clades were included in the analyses, but those found exclusively in birds of other orders were excluded. The alignments for the *Haemoproteus* and *Leucocytozoon* spp. trees contained 47 and 43 lineages, 8 and 13 of which, respectively, were detected in the present study. Sequences of *Haemoproteus columbae* hCOLIV03 and *Leucocytozoon toddi* lBUBT2 were used as outgroups for their respective genus trees. The alignments were trimmed to 474 bp, removing the first and last two bp. The best-fit substitution models based on the Akaike Information Criterion were evaluated using IQ-TREE version 2.1.3 (downloaded at http://www.iqtree.org/, accessed on 24 August 2022) [51], resulting in GTR+G+I and TN92+G for the *Haemoproteus* and *Leucocytozoon* alignments. Maximum Likelihood (ML) trees were calculated with MEGA v.7.0.26 (downloaded at https://www.megasoftware.net/, accessed on 24 August 2022) [52] using the latter models and performing 1000 bootstrap replicates each. Bayesian Inference (BI) trees were calculated with MrBayes v.3.2.2 (downloaded at https://nbisweden.github.io/MrBayes/, accessed on 24 August 2022) [53]. As MrBayes offers fewer models than IQ-TREE, the model GTR+G was used instead of TN92+G for the *Leucocytozoon* tree. The BI analyses were run for 5 million generations (2 runs with 4 chains, one of which was heated), sampling every thousandth tree. The first 25% of the trees were discarded as burn-in and a majority rule consensus tree was calculated from the remaining 3750 trees. The trees were visualized with Figtree v.1.4.4 (http://tree.bio.ed.ac.uk/software/figtree/, accessed on 7 July 2022) and finalized with Adobe Illustrator CC v.2015 (Adobe Inc., San José, CA, USA).

### 2.3. Histological Examination

Sections of 2 µm were cut from formalin-fixed paraffin-embedded (FFPE) samples, mounted on glass slides, air-dried, stained with hematoxylin–eosin (HE), and covered with coverslips. Detailed histological procedures were described in [54]. Microscopic examinations were performed using an Olympus BX51 microscope (Olympus Europa, Hamburg, Germany) equipped with an Olympus DP71 digital camera and the image software Olympus cellSens Entry. Acquired microphotographs were adjusted for brightness and contrast and assembled in Adobe Photoshop CC 2022 (Adobe, San José, CA, USA). Based on the microphotographs, measurements of exo-erythrocytic stages were performed using the ImageJ-based imaging processing package Fiji (Image J 1.53c, National Institutes of Health, Bethesda, MD, USA, downloaded at https://imagej.net/software/fiji, accessed on 24 August 2022), [55]. Exo-erythrocytic meronts were measured by their largest diameter. Unless otherwise stated, measurements are presented as mean ± standard deviation, followed by the number of measurements in parentheses.

### 2.4. Chromogenic In Situ Hybridization

CISH was applied to tissue sections of all infected birds for which tissue samples were available, using previously established probes and protocols [14,15]. In short, FFPE-tissue sections were deparaffinized and subjected to proteolytic treatment with proteinase K (Roche, Basel, Switzerland) in Tris-buffered saline. For hybridization, the sections were incubated overnight with the digoxigenin-labeled genus-specific probes. After hybridization, sections were incubated with anti-digoxigenin-AP Fab fragments (Roche) at a concentration of 1:200 to detect the digoxigenin-labelled hybrids. The color substrates 5-bromo-4-chloro-3-indolyl phosphate (BCIP) and 4-nitro blue tetrazolium chloride (NBT) (Roche) were used for visualization of the parasite-probe hybrids. Tissue sections from deceased birds containing infections with parasites of each genus were used as controls for every CISH procedure. In case of co-infections with parasites of different genera, several tissue sections were separately incubated with the relevant probes.

For labeling parasites belonging to the genera *Plasmodium*, *Haemoproteus*, and *Leucocytozoon,* the probes Plasmo18S_1, Haemo18S_1, and Leuco18S_1, which target the *18S* ribosomal RNA of haemosporidians of the respective genera, were used. For the detection of *Leucocytozoon* parasites belonging to the lineage lSTAL5, the lineage-specific probe STAL5-18S (5′-GACCGAAATCAATATATATGAAATACACA-3′) was designed based on alignments of previously published *18S* ribsosomal DNA sequences of diverse avian haemosporidians [56]. The probe was used at a concentration of 10 ng/100 µL. Cross-reactions of this probe were ruled out by negative CISH results when applied to samples containing other *Leucocytozoon*, *Plasmodium*, or *Haemoproteus* lineages, confirming its specificity.

### 2.5. Immunohistochemistry

For immunohistochemical staining of smooth muscle cells present in the middle layer of blood vessel walls, a mouse monoclonal anti-human smooth muscle actin antibody clone 1A4 (M0851, Dako, Agilent Technologies, Vienna, Austria), was used. This antibody detects the α-smooth muscle actin isoform expressed in vascular smooth muscle cells and capillary pericytes in vertebrates [57,58]. Immunohistochemistry was performed with the LabVision Autostainer 360 (Histocom, Wr. Neudorf, Austria). First, 2 µm tissue sections were deparaffinized and rehydrated. For antigen retrieval, sections were treated with citrate buffer (pH 6.0) for 20 min at 97 °C. To minimize unspecific background staining, endogenous peroxidase activity was blocked with 3% H_2_O_2_ for 5 min at room temperature. A protein block (Ultravision Protein Block, Fisher Scientific, Vienna, Austria) was applied for 10 min to prevent unspecific binding of the primary antibody. Then, sections were successively incubated for 30 min with the primary antibody diluted to a concentration of 1:500, and an HRP-labeled polymer conjugated secondary antibody (BrightVision Poly-HRP-anti mouse rabbit IgG, ImmonoLogic, Duiven, Netherlands). After each step, slides were washed with Tris-buffered saline with Tween 20 (TBS; Fisher Scientific). Finally, chromogenic detection was carried out for 5 min using 3,3′-diaminodbenzidine as a substrate (Lab Vision DAB Plus Substrate Staining System, Fisher Scientific), and sections were counterstained with Mayer’s hematoxylin (Lab Vision, Fisher Scientific) 1:30 for 1 min, dehydrated, and mounted using Neo-Mount (Merck, Darmstadt, Germany).

### 2.6. Ethical Statements

Ethical review and approval were waived for this study by the Animal Welfare Committee of the Nature Research Centre (protocol No. GGT 5) due to the fact that none of the studied individuals were harmed or experienced any suffering in regard to this study. The histological material was collected from individuals deceased due to reasons unrelated to the conduction of the present study. The collection of blood samples was approved by the institutional ethics and animal welfare committee and the national authority according to §§ 26ff. of Animal Experiments Acts, Tierversuchsgesetz 2012—TVG 2012, Austria (BMWFW-68.205/0036-WF/V/3b/2017).

## 3. Results

### 3.1. Molecular Diagnostics

PCR-based analysis showed that 89 (73.6%) of 121 owls were infected with haemosporidian parasites, showing prevalence of 1.7%, 47.1%, and 43.8% for parasites of the genera *Plasmodium*, *Haemoproteus,* and *Leucocytozoon*, respectively (Table 1). Co-infections with parasites from the same or a different genus were found in 40 (31.1%) of all tested owls. Of the genus *Haemoproteus*, four previously recorded and two newly discovered lineages were detected (Table 1). Additionally, the *Haemoproteus belopolskyi* lineage hARW1, previously reported from Sylviidae and Turdidae, was found in one owl (Table 1). Of the genus *Leucocytozoon*, nine previously reported and five new lineages were determined (Table 1). Finally, of the genus *Plasmodium,* the species *Plasmodium relictum* (lineage pSGS1) and *Plasmodium circumflexum* (lineage pTURDUS1) were detected (Table 1).

**Figure 1:**
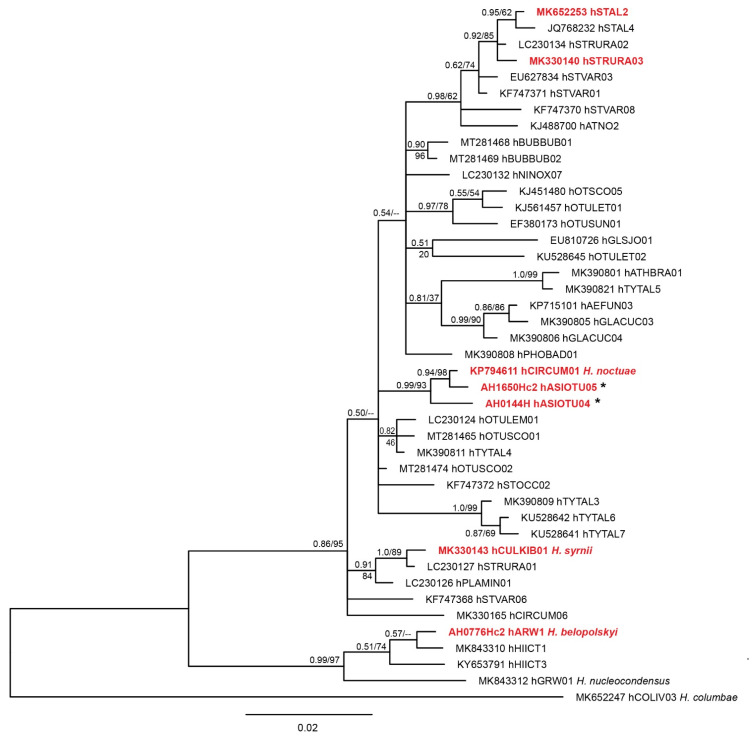
Bayesian Inference tree of 47 partial (474 bp) *Haemoproteus cytB* lineages. Lineages found in the present study are presented in red bold font. All but one lineage (hARW1) clustered within a clade featuring multiple lineages from owls. The latter (regular letters) were included in the analyses, but approximately 100 lineages from non-strigiform birds clustering in this clade were excluded a priori. For comparison, three lineages closely related to lineage hARW1 were also included. A sequence of *Haemoproteus columbae* hCOLIV03 was used as outgroup. Lineages found for the first time are marked with an asterisk. Bayesian inference posterior probabilities and Maximum-likelihood bootstrap values are indicated at each node. The scale bars indicate the expected number of substitutions per site according to the model of sequence evolution applied. GenBank accession numbers are provided, followed by lineage names according to MalAvi database (http://130.235.244.92/Malavi/, accessed on 6 July 2022). In case the lineages were linked to morphospecies, the taxon name is indicated.

Molecular phylogenetic analysis placed the previously known lineages (except hARW1), and the new *Haemoproteus* lineages hASIOTU04 and hASIOTU05 in a clade with other lineages of this genus, which were previously reported from owls (Figure 1). Within this clade, phylogeny is not fully resolved; however, the two new *Haemoproteus* lineages clustered together with hCIRCUM01, a lineage previously identified as *Haemoproteus noctuae*. The *H. belopolskyi* lineage hARW1 reported in one *Strix uralensis* in the present study clustered in a separate clade with other lineages typically reported from passerine birds.

**Figure 2:**
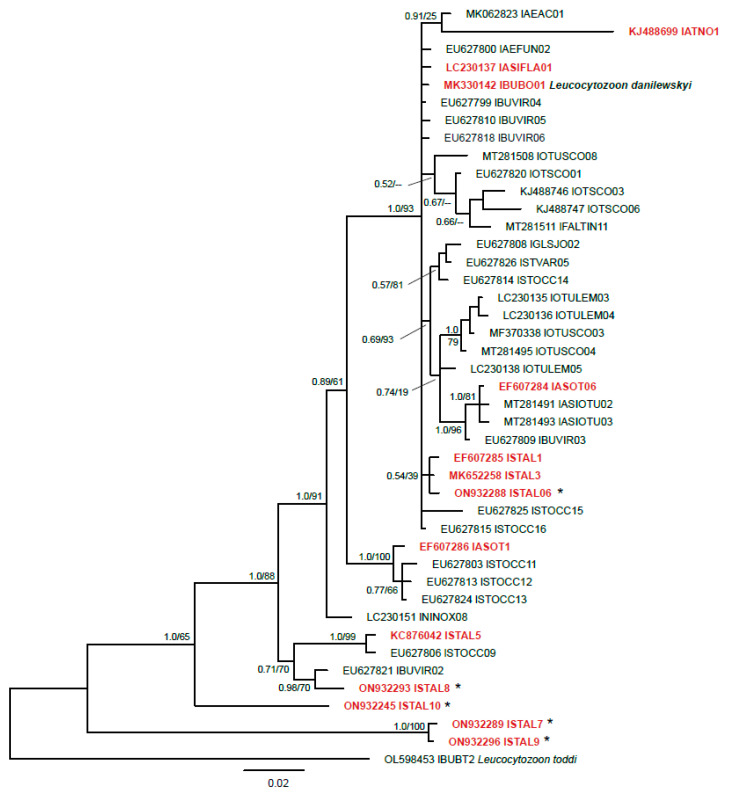
Bayesian Inference tree of 43 partial (474 bp) *Leucocytozoon cytB* lineages (474 bp). Lineages found in the present study are provided in red bold letters. Most lineages (excluding lSTAL7, lSTAL9, and lSTAL10) clustered in a clade featuring multiple lineages from owls and other birds. For comparison, all owl parasite lineages clustering in this clade were included but approximately 40 lineages from non-strigiform birds were excluded a priori. A sequence of *Leucocytozoon toddi* lBUBT2 was used as outgroup. Lineages found for the first time are marked with an asterisk. Bayesian inference posterior probabilities and Maximum-likelihood bootstrap values are indicated at each node. The scale bars indicate the expected number of substitutions per site according to the model of sequence evolution applied. GenBank accession numbers are provided, followed by lineage names according to MalAvi database (http://130.235.244.92/Malavi/, accessed on 6 July 2022). In case the lineages were linked to morphospecies, the taxon name is indicated.

Molecular phylogenetic analysis placed the new *Leucocytozoon* lineages (except for lSTAL7 and lSTAL9) in a clade with other lineages of this genus that were previously reported from owls (Figure 2). Except for *Leucocytozoon danilewskyi* lBUBO01, no other lineages in this clade have been linked to morphospecies. The lineages lSTAL7 and lSTAL9 formed a sister clade to the previously reported lineages. The smallest genetic distance between the latter two *cytB* lineages and any previously reported *Leucocytozoon* lineages were 14.4% and 14.6%, respectively.

### 3.2. Histological Diagnostics

In eight of the 51 owls screened by CISH, exo-erythrocytic stages of avian haemosporidians were found, including five *S. aluco*, two *S. uralensis,* and one *A. otus* (Table 2). In the remaining individuals, CISH signals were either absent or confined to larger blood vessels, consistent with the blood stages of the parasites. *Haemoproteus* sp. exo-erythrocytic meronts were found in one *S. uralensis* infected with *H. syrnii* (lineage hSTAL2). In regard to *Leucocytozoon* sp., exo-erythrocytic meronts of three morphologically uncharacterized lineages were found, including lASOT06 in one *A. otus*, lSTAL7 in one *S. uralensis*, and lSTAL5 in two *S. aluco*. Exo-erythrocytic meronts of three unresolved (due to co-infections) lineages of *Leucocytozoon* were reported in three individuals of *S. aluco*. Results of the analysis of the HE-stained histological sections coincided with the results of CISH.

#### 3.2.1. Description of *Leucocytozoon* Exo-Erythrocytic Stages

**Figure 3:**
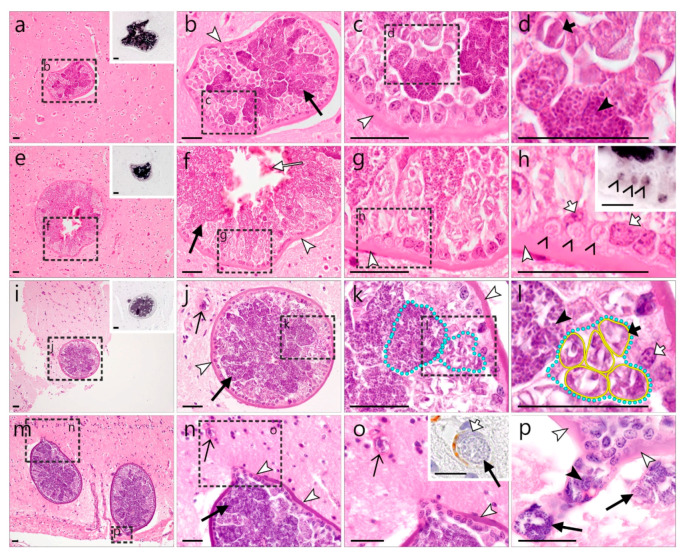
*Leucocytozoon* sp. (lineage lSTAL5) meronts in the brain of a tawny owl (*Strix aluco*). Several clusters of meronts were located in different brain areas (**a**–**o**) and identified by CISH using a *Leucocytozoon* genus- and a lSTAL5 lineage-specific probe (see inserts (**a**,**e**,**h**,**i**)). Each cluster contained numerous young (light basophilic) and mature (dark basophilic) meronts ((**b**,**f**,**j**,**n**), black arrows) and was covered by a thick eosinophilic wall (white arrowheads), the inside of which was lined by epithelial-like, cuboid host cells, likely of endothelial origin. Sometimes, intermingling erythrocyteys were observed ((**f**), white arrow). Individual meronts showed the formation of roundish cytomeres (**l**), giving the meronts a grape-like appearance (see (**k**,**l**), yellow line—cytomere outline, blue dotted line—meront outline). Developing meronts comprised multiple roundish cytomeres, which were separated by vacuolic spaces (e.g., (**d**,**l**) short black arrows), whereas mature meronts contained numerous round merozoites ((**d**,**l**) black arrowheads). Tiny, intracellular early meronts were observed in vacuoles within host cells lining the wall ((**h**), open arrowheads) and were labeled by the *Leucocytozoon* probe ((**h**), insert). Notably, blood capillaries located close to the observed meront clusters also contained small meronts ((**j**,**n**,**o**,**p**), open arrows). Figure (**o**) shows a higher magnification of a small meront developing close to the large structure (arrows) with the insert picture demonstrating its intraendothelial location (note the endothelial host cell nucleus— short, white arrow, and the immunohistochemical labeling of actin—brown diaminobenzidine (DAB), a marker for smooth muscle cells and pericytes around microvessels). Protrusions of the wall bordering the meront clusters towards nearby microvessels (as seen in (**o**,**p**)) indicate a vascular connection. Long black arrow (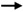)—meront, short black arrow (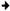)—cytomere, black arrowhead (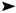)—merozoite, open arrowhead (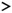)—intracellular young parasite stages, open arrow (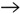)—meront containing capillary, long white arrow (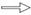)—erythrocytes, short white arrow (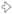)—host cell nucleus, white arrowhead (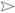)—wall. All scale bars are 25 µm.

In the brains of two *Strix aluco*, multiple clusters of morphologically similar meronts were found (Figure 3). The parasite structures were located in different areas of the brain (Figure 3a,e,i,m) and were identified as *Leucocytozoon* sp. lineage lSTAL5 by CISH using a *Leucocytozoon* genus- and a lSTAL5 lineage-specific probe (Figure 3 inserts in a,e,h,i). Each cluster comprised numerous individual meronts, occasionally intermingling erythrocytes, and was entirely bordered by a thick eosinophilic wall-like structure. An epithelium-like layer of cuboid host cells, most likely of endothelial origin, lined the inside of the wall. Meront clusters covered by the wall varied in size and shape, measuring up to 300 µm in their largest diameter (236.5 ± 57.0 µm, *n* = 5). The overall shapes of the clusters were mostly roundish to oval; however, sometimes they appeared irregular in adjacent histological sections (for example, compare the HE-stained and CISH-labeled structures in Figure 3a,e). Generally, individual meronts inside the clusters were not well delimitable, but seemed to comprise multiple roundish cytomeres, giving the meronts a grape-like appearance (e.g., Figure 3c,d,k,l). Young cytomeres appeared as light basophilic structures and were marked by vacuolic spaces, while mature meronts were strongly basophilic and contained numerous round merozoites (Figure 3d,l). Within the clusters, meront development was asynchronous, i.e., growing and mature meronts were located side by side, although more developed meronts seemed to gather centrally of the cluster (note the strong basophilic central areas in the meront clusters in Figure 3b,f,j,m), whereas growing stages were closely associated with the wall border (less basophilic areas beneath the eosinophilic wall). Generally, host cells or host cell nuclei were not discernable around mature meronts. By contrast, tiny young parasite stages were located intracellularly in cells with readily visible nuclei; the parasites seem to be located in vacuoles of the host cells lining the wall (Figure 3h). Notably, single small meronts were sometimes found in close association to the large meront clusters (Figure 3j,n,o). These small meronts were located in endothelial cells of brain capillaries, as confirmed by positive immunohistochemical staining of actin present in smooth muscle cells and pericytes around microvessels (Figure 3o). Occasionally, protrusions of the wall bordering the large meront clusters, towards the smaller microvessels, were observed, indicating a vascular connection between these structures (Figure 3n–p).

Occasionally, mild reactive gliosis was observed around the meront cluster-bordering wall. Similarly, brain vessels found in close proximity to the meront clusters showed perivascular inflammation (Figure S1).

**Figure 4:**
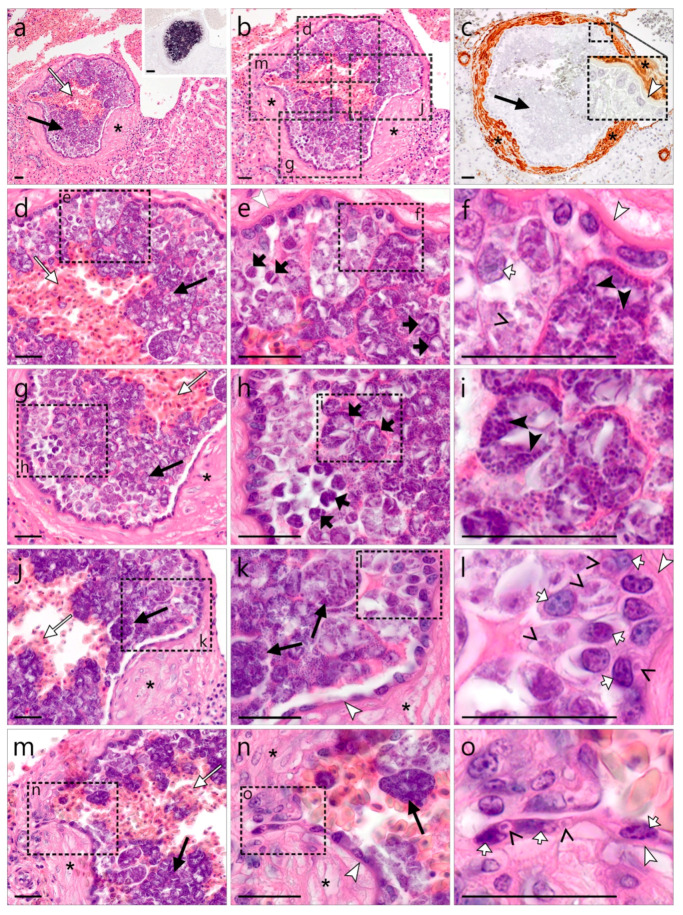
*Leucocytozoon* sp. (lineage STAL5) meronts in the kidney of a tawny owl (*Strix aluco*). A cluster of meronts morphologically similar to those observed in the brain was located in the renal cortex of the same individual and identified by CISH using a lSTAL5 lineage-specific probe (a, insert). The meront clusters were covered by an entire thick eosinophilic wall-like structure, encompassed by multiple layers of smooth muscle cells ((**a**,**b**), asterisks), as demonstrated by immunohistochemical labeling of actin—the smooth muscle cell marker ((**c**), asterisk—brown DAB staining)—indicating their intravascular location. Numerous erythrocytes were observed in the central lumen of the meront cluster ((**a**), long, white arrow). Subfigures (**d**,**g**,**j**,**m**) represent magnifications of the four different areas of the meront cluster indicated by rectangles in (**b**). The meronts appeared grape-like due to the formation of roundish cytomeres ((**e**,**h**), short black arrows). Young meronts were closely associated with the host cells beneath the wall and characterized by slight basophilic staining and loosely distributed large nuclei ((**f**), open arrowhead). Note the host cell nucleus in a growing meront ((**f**), short white arrow). Mature meronts were mostly observed in the center of the cluster and contained numerous small, round, and strongly basophilic merozoites ((**f**,**i**), black arrowheads). The inside of the eosinophilic wall was lined by epithelial-like squamous to cuboidal cells, likely attributable to endothelium. Many of these cells contained small young parasite stages within a vacuole ((**l**,**o**), open arrowheads). Note the transition from squamous, endothelial-like host cells along the wall in areas with little parasite disturbance ((**k**), bottom left) over to cuboidal cells in areas with extensive parasite involvement ((**k**), top right). In the middle left area of the entire host-parasite cell complex, a loop-like evagination was observed, likely representing a vascular branch (**m**,**n**). High magnification of this area reveals intracellular parasite stages in endothelial cells ((**o**), open arrowheads). Long black arrow (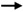)—meront, short black arrow (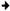)—cytomere, black arrowhead (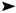)—merozoite, open arrowhead (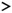)—intracellular young parasite stages, long white arrow (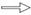)—erythrocytes, short white arrow (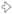)—host cell nucleus, white arrowhead (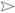)—wall, asterisk (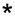)—smooth muscle layer. Scale bars are 25 µm.

In one of the two *S. aluco* showing *Leucocytozoon* sp. lSTAL5 meronts in the brain, a single cluster of morphologically similar meronts was found in the renal cortex of the kidney (Figure 4). As in the brain, the cluster contained numerous asynchronously growing and maturing meronts, enclosed by a thick eosinophilic wall-like structure. The observed cluster showed a maximum diameter of 312 µm and was more or less roundish in shape, although loop-like protrusions of the wall were observed in adjacent histological cuts of the structure (Figure 4m–o). Around the eosinophilic wall, multiple layers of smooth muscle cells resembling the prominent smooth muscle layer in the wall of arteries were observed. Positive immunohistochemical staining of these cells with actin—a smooth muscle marker—confirmed their origin and the intravascular location of the detected meronts (Figure 4c). In the central lumen of the meront-containing vessel, numerous erythrocytes were present (Figure 4a). The meronts showed grape-like shapes due to the formation of roundish cytomeres (Figure 4e,h). As in the clusters found in the brain, growing and mature meronts were located side by side, albeit mature stages gathered more centrally, distinguishable by their strong basophilic appearance and the presence of merozoites. Growing meronts were characterized by slight basophilic staining and loosely distributed, large nuclei. Often, a host cell nucleus was seen in growing meronts (Figure 4f). Mature meronts contained numerous small, round, strongly basophilic merozoites (Figure 4i). The entire inside of the eosinophilic wall was lined by epithelial-like squamous to cuboidal host cells, likely attributable to the endothelium. Many of these cells contained small, young parasite stages within a vacuole (Figure 4l,o). Similar to the wall protrusions observed in the clusters located in the brain, a loop-like evagination of the wall and endothelial-like cells were noticed, likely representing a vascular branch (Figure 4m,n).

Besides the above-described meronts detected in the brains and kidneys, several lSTAL5 meronts were also found in the heart muscle of one *S. aluco*. The developing meronts were found in two locations in the myocardium and measured approximately 8.2 ± 3.35 µm (*n* = 2).

**Figure 5:**
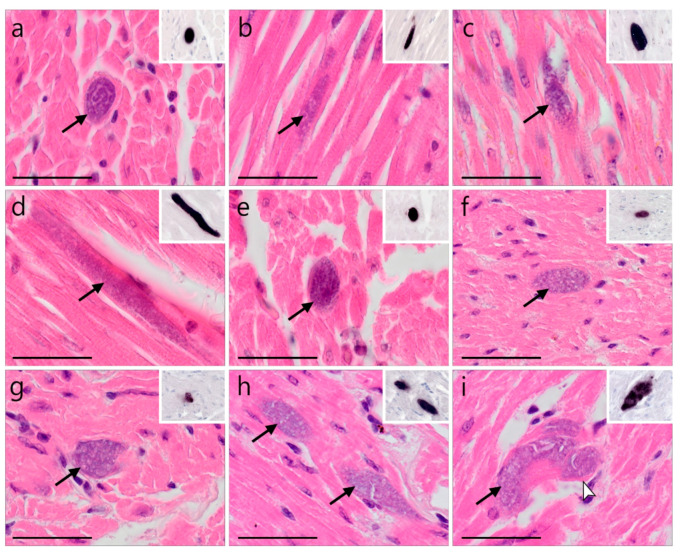
*Leucocytozoon* spp. meronts in the heart muscles of a long-eared owl (*Asio otus*) and three tawny owls (*Strix aluco*) infected with *Leucocytozoon*. Meronts found in HE-stained sections were identified by CISH using a *Leucocytozoon*-specific probe (inserts (**a**–**i**)). (**a**,**b**) Meronts in cardiomyocytes of a *A. otus* infected with lineage lASOT06 (Table 1 individual: AH1539). (**c**–**i**) Meronts found in the myocardium of three *S. aluco* infected with multiple, not further characterized *Leucocytozoon* lineages (Table 1 individuals: (**c**)—AH1957, (**d**,**e**)—AH0410, (**f**–**i**)—ZA21/18). Generally, meronts found in the heart were roundish or oval (**a**,**e**–**i**) but appeared elongate in longitudinal cuts of the myocardium (**b**,**d**). Most meronts seemed to be growing as merozoites were not discernable, whereas mature/nearly mature meronts were only exceptionally observed (**e**), indicating asynchronous development. Occasionally, a thin eosinophilic wall-like structure around the meront was visible ((**i**), white arrowhead). Long black arrow (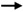)—meront, white arrowhead (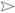)—wall. Scale bars are 25 µm.

In one *A. otus*, several meronts of *Leucocytozoon* sp. lineage lASOT06 were found in the myocardium (Figure 5a,b). They were either roundish or elongate in shape and 17.4 ± 3.8 µm (*n* = 3) in their largest diameter. They were growing, as merozoites were not yet identifiable.

Plenty meronts of other, non resolved on the lineage level, *Leucocytozoon* parasites were also found in the heart muscles of three *S. aluco* infected with multiple parasite lineages (Figure 5c–i). Generally, the meronts detected in the myocardium of the three individuals were diffusely distributed over the entire section of the heart muscle. They were mostly roundish to oval but appeared elongate in longitudinal cuts of the myocardium. Most meronts seemed to be growing, whereas mature meronts, characterized by the presence of numerous merozoites, were only exceptionally observed (Figure 5e). Occasionally, a thin eosinophilic wall-like structure was visible around the meronts (Figure 5i). The sizes of the meronts found in the three individuals were comparable, measuring 30.5 ± 15.1 µm (*n* = 6) (Figure 5c), 39.0 ± 32.9 µm (*n* = 3) (Figure 5d,e), and 22.0 ± 17.8 µm (*n* = 25) (Figure 5f–i).

Finally, two *Leucocytozoon* sp. (lineage lSTAL7) meronts were found in the kidney of S. *uralensis*. These meronts were only detected by CISH, and they seemed to be mature based on the dotted pattern of the CISH signal, indicating the presence of multiple merozoites. The meronts were small, measuring 11.8 ± 3.53 µm (*n* = 2).

#### 3.2.2. Description of *Haemoproteus* Exo-Erythrocytic Stages

**Figure 6:**
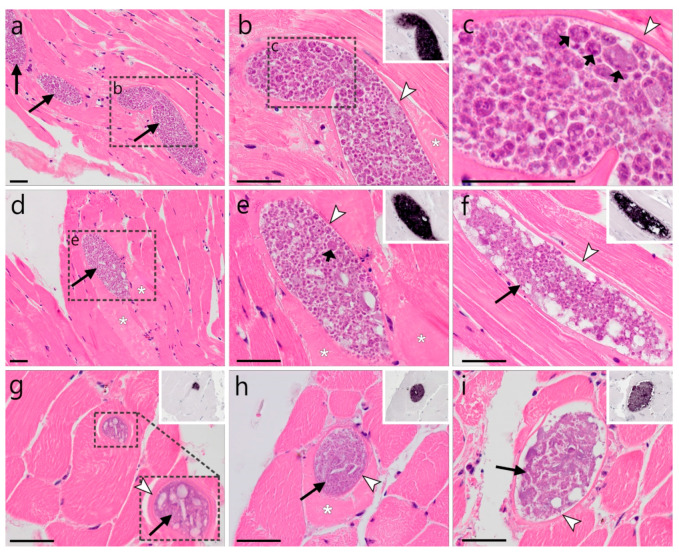
*Haemoproteus syrnii* (lineage hSTAL2) megalomeronts in the skeletal muscle of a Ural owl (*Strix uralensis*). Multiple growing megalomeronts (long arrows) were detected by HE-staining and CISH using a *Haemoproteus*-specific probe ((**b**,**e**–**i**), inserts). The megalomeronts were located in muscle fibers and were covered by a thin eosinophilic wall (arrowheads). Maturity of the stages varied among megalomeronts, indicating asynchronous development. Young megalomeronts were characterized by a basophilic more or less uniform content interspersed with vacuolic and cleft-like spaces (**g**,**i**). Megalomeronts more advanced in their development showed roundish cytomeres of varying sizes, which contained plenty of cytoplasm and large basophilic nuclei ((**a**–**f**), short arrows). Note that the infected muscle fibers, particularly those containing more advanced megalomeronts, were marked by hyaline degeneration with a loss of myofiber striation and hypereosinophilia ((**b**,**d**,**e**,**h**), asterisks). Long black arrow (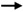)—megalomeront, short black arrow (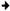)—cytomere, white arrowhead (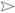)—wall, asterisk (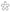)—necrosis. Scale bars are 50 µm.

Multiple *Haemoproteus syrnii* (lineage hSTAL2) megalomeronts were detected in the skeletal muscle of a *Strix uralensis* (Figure 6). The megalomeronts were located in muscle fibers showing oval to elongated shapes, depending on whether the parasite was sectioned longitudinally or transversally. All detected megalomeronts were covered by a thick eosinophilic wall, showing growing cytomeres of varying maturity. Early cytomeres were characterized by a basophilic content interspersed with vacuolic or cleft-like spaces (Figure 6g), whereas megalomeronts more advanced in their development showed roundish cytomeres containing plenty of cytoplasm and large basophilic nuclei (Figure 6a–f). Megalomeronts measured a maximum of 148.5 µm in their largest diameter (80.9 ± 44.6, *n* = 8). Cytomeres within individual megalomeronts varied in size, measuring 5.1 ± 2.02 µm (*n* = 54). Infected muscle fibers, particularly containing megalomeronts more advanced in their development, frequently showed signs of necrosis. They were marked by a loss of myofiber striation and hypereosinophilia, indicating hyaline degeneration of the infected cells (Figure 6b,d,e,h).

## 4. Discussion

Despite increasing knowledge and accumulating studies on avian haemosporidians parasites in wild birds, owls remain among the least explored birds, particularly regarding the exo-erythrocytic development of the parasites’ life cycle. Using a combined molecular, genetic, and histological approach, the present study aimed to investigate avian haemosporidian infections in wild owls from Europe. The major findings of this study are (i) new insights into the genetic diversity of avian haemosporidian parasites infecting owls, (ii) the discovery of a new mode of exo-erythrocytic development of *Leucocytozoon* (subgenus *Leucocytozoon*) parasites (lineage lSTAL5), and (iii) the first report of megalomeronts of the widespread owl parasite *H. syrnii* (lineage hSTAL2) in a naturally infected *S. uralensis*.

Former extensive studies using microscopic examination of blood films showed that owls are common hosts of haemosporidian parasites in the Americas, Europe, Asia, and Africa [28,59,60,61,62]. A high prevalence of haemosporidian parasites (73.6%) was also found in the present study (Table 1). This finding coincides with previously published data, including molecular screening of haemosporidians in owls [31,32,33,34,36,42,63], and supports the notion that birds of the order Strigiformes are prone to haemosporidian infections. However, it should be pointed out that the bird sample sizes in this study differed strongly between the investigated host species. While some common host species were sampled quite extensively (*A. otus n* = 30; *S. aluco n* = 39; *S. uralensis n* = 29), several other species were underrepresented (for example, *A. flammeus n* = 1; *A. noctua n* = 3) (Table 1). Thus, generalizations on the prevalence of haemosporidians on species levels of Strigiformes based on the data from this study should be avoided. However, comparing the prevalence from the well-sampled species of this study (*A. otus* prevalence 70%; *S. aluco* 87.2%; *S. uralensis* 82.8%) to previously published data with a prevalence of 82% in *A. otus* [32], 60% in *S. aluco* [36], and 64.3% in *S. uralensis* [31], the idea of high susceptibility of the aforementioned host species to haemosporidian pathogens is confirmed.

Owls are often infected by multiple species of avian haemosporidian parasites. In the present study, approximately one-third of the tested owls harbored co-infections. While co-infections are common in the wild, their effects on the host remain poorly investigated. Recently, Palinauskas et al. [64] experimentally showed how haemosporidian parasites of two different species (*Plasmodium relictum* and *P. elongatum*) interact during co-infection. The intensity of *Plasmodium elongatum* parasitemia was enhanced by the presence of *P. relictum* during co-infection, while the parasitemia of *P. relictum* remained the same and the virulence of the co-infections was similar to that of the more virulent parasite *P. elongatum*. The authors also reported changes in the profile of infection and the mechanisms causing mortality. Similarly, a study from the United Kingdom [65] reported fatal co-infection with *Haemoproteus noctuae* and *Leucocytozoon danilewskyi* (syn. *Leucocytozoon ziemanni*) in *Bubo scandiaca*. Although the virulence of *H. noctuae* and *L. danilewskyi* has been insufficiently investigated, the authors suggested that the parasites’ interaction during the co-infection resulted in the death of the infected individuals. The effects of other co-infections, including those often found in naturally infected owls, remain unclear and should be further investigated taking into consideration the most frequent host–parasite species combinations observed in wild birds.

Two new genetic lineages of *Haemoproteus* and five new lineages of *Leucocytozoon* were reported for the first time in this study. Similarly, Barino et al. [42] reported six new lineages and Pornpanom et al. [66] 17 new lineages of avian haemosporidians in owls. Such high numbers of newly discovered lineages suggest that the diversity of avian haemosporidians infecting owls is vastly underestimated. This could be attributed to the fact that wild owls are rarely sampled due to their lifestyle, difficulties in accessing these birds, or bioethical permit issues. The lack of understanding of the biodiversity of parasites developing in owls must be addressed as owls seem to suffer from haemosporidian infections and might serve as reservoir hosts and facilitate the spread of various haemosporidian infections.

Two of the newly reported *Leucocytozoon* lineages are quite peculiar because they are genetically distant from all known lineages. The *cytb* sequences of lineages lSTAL7 and lSTAL9 differ at least 14.4% and 14.6% from any previously reported *Leucocytozoon* lineages. While the genetic distance of >14% may seem quite high, when considering lineages belonging to parasites within the same genus of avian haemosporidians, similarly high genetic distances have been reported among lineages attributed to the genus *Leucocytozoon* (subgenus *Leucocytozoon*) by Lotta et al. [67]. At present, the genus *Leucocytozoon* consists of two subgenera—*Leucocytozoon* and *Akiba*. The reported high genetic variation within the genus *Leucocytozoon* ([67,68]; present study) suggests a more complex phylogeny than currently assumed, and potentially, in the future, the genus *Leucocytozoon* should be addressed from the taxonomical point of view. Further research on the life cycles, exo-erythrocytic development, blood-stage morphology, vector biology, and genetic diversity is needed to fully understand the phylogenetic relationships within this diverse group of organisms. Unfortunately, the lack of available blood films in this study prevents a more in-depth analysis of this parasite group. However, as the two new lineages were reported from a total of five individuals of *S. aluco* in Europe, this bird species could be suggested for targeted sampling of the respective lineages in future investigations.

One of the most interesting and important findings of this study was the discovery of exo-erythrocytic meronts of *Leucocytozoon* sp. (lineage lSTAL5) in the brains and kidneys (Figure 1 and Figure 2) of naturally infected *S. aluco*. At first glance, the observed structures, based on their morphology, with the exception of the lack of a central body (enlarged host cell nucleus), appeared similar to megalomeronts of *Leucocytozoon* spp., because the formation of a structure similar to a capsular-like wall was readily visible and parasite stages enclosed by the wall resembled cytomeres. Megalomeronts of *Leucocytozoon* sp. have also previously been reported in the brain of the vertebrate hosts [69], which can be explained by the parasites’ ability to develop in macrophages in various internal organs [3]. In theory, the central body might not be visible in the histological section, depending on its location within the megalomeront. However, the available histological material of our study showed that these observed structures are not megalomeronts, but rather aggregations of meronts developing within a blood vessel lumen. Firstly, the structures similar to the capsular-like wall were immunopositive for smooth muscle actin—a marker for smooth muscle cells and pericytes around blood vessels (see inserts in Figure 3o and Figure 4c). Secondly, some blood capillaries close to the observed meront clusters also contained small meronts, located in endothelial cells (Figure 3j,n–p, open arrows). Notably, protrusions of the wall bordering the large meront clusters towards the smaller capillaries were noticed, indicating a vascular connection between them. In other words, a branching blood vessel with exo-erythrocytic meronts was observed. Thirdly, the entire inside of the capsular-like wall structure was lined with a layer of host cells, which resembled endothelial cells. Unfortunately, attempts to immunolabel endothelial cells using commonly applied endothelia-specific markers failed in FFPE avian tissues (unpublished data), hence these cells could not be immunohistochemically determined. Importantly, multiple host cells contained lSTAL5-CISH-positive signals, likely representing very young parasite stages (merozoites, trophozoites, or early meronts) (Figure 3h). Whether these cells were infected or actively phagocytized the parasite stages remain unclear, but they seemed to detach from their original location as the parasites grew, and flow into the lumen of the blood vessel, where exo-erythrocytic meronts then developed further, resulting in the presence of meront clusters. Finally, erythrocytes were visible inside some of the structures (Figure 3f and Figure 4a,b,d,e,g,j,m). Such inclusions of erythrocytes are morphologically similar to ruptures of true mature megalomeronts, followed by infiltration of blood cells as was described in the case of *Haemoproteus pastoris* in *Sturnus vulgaris* by Duc et al. [25]. However, in the context of the previously described traits, it is more likely that, in our case, the observed erythrocytes were part of the normal blood flow within the vessel, rather than a pathology. This atypical development of “free” meronts within the lumen of blood vessels is an exciting finding illustrating an unknown mode of exo-erythrocytic development in *Leucocytozoon* parasites and possibly other haemosporidian parasites. In fact, the development of exo-erythrocytic stages, visually similar to that reported in the present study, was described in *Leucocytozoon caulleryi* of the subgenus *Akiba* of the genus *Leucocytozoon*, a pathogen parasitizing chicken in Southeast Asia [3]. However, in the case of *L. caulleryi,* the mode of exo-erythrocytic development is different: growing megalomeronts develop in endothelial cells, which rupture, and the developing cytomeres escape from them and finish their development and maturation extracellularly in adjacent organ tissues, in which clusters of developing cytomeres are often visible. However, in the present study, meronts of *Leucocytozoon* sp. lSTAL5, belonging to the subgenus *Leucocytozoon*, were identified inside the intact blood vessels. The exact mechanism and biological function of this development are unclear. The authors raise the hypothesis that sporozoites and/or merozoites invade endothelial cells of capillaries and are pushed out into the vessel lumen as the parasites grow. Merogony completes in the vessel lumen and numerous merozoites invade the most closely located cells, resulting in asynchronous development of meronts in the same cluster of parasites. Further studies are needed to confirm this hypothesis.

The absolute sizes of the observed *Leucocytozoon* sp. lSTAL5 intravascular clusters and the extent to which the blood vessels were affected remain elusive, because only a couple of serial sections could be analyzed during this study. While this limits the interpretation of findings with regard to how much these stages affected host health, the observed gliosis around infected vessels, indicating a tissue reaction, together with the fact that some vessels were completely blocked by the developmental stages, suggest detrimental effects on cerebral and renal blood flow, which can hardly be well tolerated by the host.

In the present study, megalomeronts of *H. syrnii* (lineage hSTAL2) were discovered for the first time and were reported in one naturally infected *S. uralensis* originating from Lithuania. Previously, Barino et al. [42] reported similar results on the exo-erythrocytic development of a *Haemoproteus* parasite from Brazil. The authors claimed that the parasite they found was *H. syrnii*, however, the provided morphological gametocyte traits of the described organism do not coincide with those of *H. syrnii* description due to the lack of volutin granules [70]. These authors likely characterized a new, previously unencountered species of *Haemoproteus*. In their study, Barino et al. [42] discovered exo-erythrocytic stages of *Haemoproteus* sp. in the skeletal muscle, lungs, and liver of the infected *Megascops choliba*. In contrast, in the present study, megalomeronts were observed in the skeletal muscle of the infected *S. uralensis.* Some megalomeront-induced necroses were observed, but mostly restricted to infected muscle fibers. Based on the reported high prevalence of *H. syrnii* in *S. uralensis*, and the presence of *H. syrnii* gametocytes in the circulation [3], it is likely that this parasite is well adapted to develop in *S. uralensis* and other owl species in such a manner as to not cause much damage to the vertebrate host. Such adaptations are common among *Haemoproteus* spp. developing in birds where co-evolution occurred [3]. Contrary to this, massive tissue damage caused by developing exo-erythrocytic stages was reported when *Haemoproteus minutus* and closely related lineages infected non-adapted Australasian and South American parrots in Europe [23,71]. *Haemoproteus minutus* megalomeronts damaged various internal organs, particularly the heart and gizzard of the infected individuals. Massive hemorrhagic lesions were reported in the deceased parrots. In these cases, *H. minutus* could not complete its development and produce invasive merozoites resulting in the absence of parasitemia but causing the death of the hosts due to exo-erythrocytic development [23]. Similar results of adapted owls serving as a reservoir host for lethal infections of owl-specific lineages to non-adapted birds were recently described in Spain [72]. *Haemoproteus syrnii* was detected in two deceased parrot individuals of *Trichoglossus haematodus* and *Psittacula cyanocephala* [72]. The same parasite was also reported from deceased *Tyto alba* and *Strix rufipes*. All four birds (parrots and owls) were housed and died in the same rehabilitation center, suggesting that local transmission might have occurred. The authors drew the conclusion that owl parasites were lethal to the infected parrots [72]. This observation is a serious call for attention as it is common to find owls in rehabilitation centers and zoos all over the world and it remains unclear whether owl-specific parasite species can cause serious pathologies and even mortality in non-adapted birds of other species. This should be taken into consideration regarding bird health measures and conservation activities. In order to understand how haemosporidian parasites developing in owls affect birds belonging to other families or even orders, and to prevent such lethal events in rehabilitation centers, zoos, and potentially the wild, it is paramount to investigate the life cycles and the diversity of these under-investigated pathogens further.

The findings of the present study support the data first published by Ilgūnas et al. [16] and later confirmed by Duc et al. [25] that *Haemoproteus* megalomeronts develop not only during abortive infections, but also during normal development of the parasites in the vertebrate host. Ilgūnas et al. [16] showed that *H. majoris* developed parasitemia and megalomeronts at the same time in infected *Parus major* and *Turdus pilaris*, while Duc et al. [25] showed the same result with *H. pastoris* in *Sturnus vulgaris.* It needs to be pointed out that in the present study, blood films were not available for analysis of erythrocytic development. Instead, positive CISH signals were observed in erythrocytes during this study, adding the *S. uralensis*–*H. syrnii* pair to the list of host–parasite associations where both megalomeronts and gametocytes develop, indicating a successful development in the host. Together with the findings of the previous studies, these results indicate a pattern of exo-erythrocytic development of haemoproteid parasites.

It remains unclear how *H. syrnii* persists in the vertebrate host over time. The *S. uralensis* investigated in the present study was found dead in August, towards the end of the vector transmission season in Lithuania, thus it is impossible to say if megalomeronts would remain in the host’s body over the winter when the transmission of parasites is absent and there is no need for the parasite to develop erythrocytic stages due to the low activity of vectors. During haemoproteid infections, only exo-erythrocytic stages can facilitate the persistence of *Haemoproteus* parasites, as these pathogens do not multiply in the blood [3]. However, in none of the histologically examined 21 *H. syrnii*-infected owls, seven of which died between the months of November and March, exo-erythrocytic stages were found. The results suggest that megalomeronts likely are not the persisting stage for *H. syrnii*. It is possible that exo-erythrocytic merozoites of *H. syrnii* invade tissue cells of a vertebrate host, but do not start multiplying until the next transmission season begins, similarly as in the case of *Plasmodium* spp. hypnozoites during human malaria [73]. Unfortunately, currently available methodologies might not be sensitive enough to detect such small stages. To identify possible dormant stages of *H*. *syrnii*, ISH-based techniques should be developed further. Interestingly, the remaining 14 *H. syrnii*-infected individuals studied in the present investigation were obtained during the months of April to October, a time when gametocytes are needed for the parasite to be able to spread, and yet no other exo-erythrocytic stages besides the described megalomeronts in one *S. uralensis* individual, were observed. Undoubtedly, this result is not conclusive as not all organs were present for the investigation; however, it shows how little the patterns of avian haemoproteid exo-erythrocytic development and persistence are understood. This gap in knowledge must be filled to better understand the biology, ecology, and pathogenicity of avian haemoproteid parasites, which is the basic information needed for the development of infection control.

Exo-erythrocytic meronts of unidentified *Leucocytozoon* lineages were observed in three individuals of *S. aluco* and in one individual of *A. otus* infected with the lASOT06 in the present study. Interestingly, these exo-erythrocytic stages were all observed in the heart muscle of the infected individuals, suggesting a similar site-development pattern. While nothing can be said about the species identity of three of the four observed meronts, the fact that the parasites tend to develop in the heart could be indicative of a possible pattern of exo-erythrocytic development of *Leucocytozoon* spp. in owls. Likewise, Harl et al. [68] reported exo-erythrocytic meronts of *Leucocytozoon* parasites exclusively in the kidney of Accipitridae birds. While general conclusions on the patterns of exo-erythrocytic development of *Leucocytozoon* spp. in owls cannot be drawn based on four investigated individuals, the findings indicate possible directions for future investigations.

Interestingly, the *Haemoproteus belopolskyi* lineage hARW1 was reported from one *S. uralensis* in the present study. This lineage has previously only been reported from birds of the families Sylviidae or Turdidae, thus the detection of the parasite in this atypical host raises questions about the infection. This incidence is likely a report of a “dead-end infection” leading to abortive development when an infected competent vector takes bloodmeal from a non-competent host and, during the process of transmission, injects sporozoites into the bloodstream of that host. The sample of this specific *S. uralensis* individual originated during the month of July when the vector season and, in turn, the transmission of haemosporidian parasites are at their peaks in Lithuania. Similar results have been reported earlier during *Leucocytozoon* infections [74]. In order to resolve such questions, reports of gametocytes would be helpful, and quality blood films should be prepared and analyzed. Unfortunately, such material was not available in this case, thus the authors can only speculate. No conclusions should be drawn on the ability of *H. belopolskyi* to develop in owls based on this single PCR-based present report.

## 5. Conclusions

Two previously unreported lineages of *Haemoproteus* and five of *Leucocytozoon* were detected during this investigation, adding new knowledge to the under-investigated genetic diversity of haemosporidian parasites infecting owls. This study discovered and described meronts of *Leucocytozoon* parasites of the lSTAL5 lineage and megalomeronts of *Haemoproteus syrnii* (lineage lSTAL2). Importantly, the mode of exo-erythrocytic meront development of the *Leucocytozoon* sp. lineage lSTAL5 was found to be different from all other known *Leucocytozoon* spp., as clusters of individual meronts were seen developing freely in the lumen of blood vessels of kidneys and/or brain causing a tissue reaction in these infection cases, hinting at possible pathological changes and negative effects on the owls’ health. The biological meaning of the atypical development in the lumen of blood vessels remains unclear and warrants further investigation. This finding broadens the understanding of the development and pathological effects of leucocytozoonoses and calls for further research of these neglected avian infections. The discovery of the megalomeronts of *H. syrnii* (lineage lSTAL2) in *S. uralensis* adds to the understanding of the development of haemoproteids, supporting to the notion that megalomeronts develop not only during abortive infections, but are also part of the regular life cycle during natural infections in competent hosts. Finally, meronts of the lASOT06 and three other unknown lineages of *Leucocytozoon* were also reported in the heart muscles of infected individuals and suggest a possible pattern of leucocytozoonosis development in strigiform birds, which should be investigated further for possible negative effects on the host health.

## Figures and Tables

**Table 1:** Haemosporidian parasites and *cytB* lineages recorded in species of Strigiformes by PCR and sequencing.

Host Family and Species	*n* Examined	Sample Origin (*n*)	*n* Positive				Identified *cytB* Lineages ^a^		
			*Plas*	*Haem*	*Leuc*	Total (%)	*Plasmodium*	*Haemoproteus*	*Leucocytozoon*
**Strigidae**									
*Asio flammeus*	1	AT	0	0	1	1 (100%)	-	-	*L.* sp. lASIFLA01 (1)
*A. otus*	30	AT (24), LT (6)	0	9	18	21 (70%)	-	*H. noctuae* hCIRCUM01 (5); *H*. sp. **hASIOTU04** (2), **hASIOTU05** (1)	*L*. sp. lASOT1 (1); lASOT06 (17),
*Athene noctua*	3	AT	1	0	1	1 (33.3%)	*P. relictum* pSGS1 (1)	-	*L*. sp. lATNO1 (1)
*Bubo bubo*	10	AT	0	4	3	5 (50%)	-	*H.* sp. hSTRURA03 (4)	*L. danilewskyi* lBUBO01 (2); *L.* sp. lSTAL3 (1)
*Glaucidium passerinum*	6	AT (1), LT (5)	0	1	2	3 (50%)	-	-	*L*. sp. lASOT06 (1), lSTAL3 (1)
*Strix aluco*	39	AT (17), LT (22)	0	23	21	34 (87.2%)	-	*H. syrnii* hSTAL2 (15), hCULKIB01 (11)	*L.* sp. lSTAL1 (1), lSTAL3 (4), lSTAL5 (4), **lSTAL6** (1), **lSTAL7** (3), **lSTAL8** (1), **lSTAL9** (3), **lSTAL10** (1)
*Strix* sp.	1	AT	0	0	0	0 (0%)	-	-	-
*S. uralensis*	29	AT (26), LT (3)	1	20	7	24 (82.8%)	*P. circumflexum* pTURDUS1 (1)	*H. syrnii* hCULKIB01 (9), hSTAL2 (14); *H. belopolskyi* hARW1 (1); *H.* sp. hSTRURA03 (1)	*L*. cf. *californicus* lCIAE02 (1); *L.* sp. lSTAL3 (5), **lSTAL7** (1), **lSTAL10** (1),
**Tytonidae**									
*Tyto alba*	2	AT (1), LT (1)	0	0	0	0 (0%)	-	-	-
Total	121		2	57	53	89 (73.6)			

AT: Austria; LT: Lithuania. *Plas*: *Plasmodium*; *Haem*: *Haemoproteus*; *Leuc*: *Leucocytozoon.* ^a^ Number of positive individuals is given in parentheses. Lineages in bold represent novel *cytb* haplotypes.

**Table 2:** Haemosporidian exo-erythrocytic stages detected in tissue sections of infected owls.

Host Species	Individual	Parasite Species and *cytB* Lineage	Infected Organs and Distribution of Meronts ^a,b^
*Asio otus*	AH1539	*L.* sp. lASOT06	heart muscle: meronts (*L*)—multifocal
*Strix aluco*	AH0145	*L.* spp. lSTAL5, lSTAL9	brain: meronts (*L*)—oligofocal
	AH0221	*H. syrnii* hCULKIB01, *L.* sp. lSTAL5	brain: meronts (*L, S*)—multifocal kidney: meronts (*S*)—focal heart muscle: meronts (*L*, *S*)—oligofocal
	AH0410	*H. syrnii* hCULKIB01, hSTAL2, *L.* sp. lSTAL5, *L.* sp. (unresolved)	heart muscle: meronts (*L*)—diffuse
	AH1957	*L.* sp. lSTAL5, *L.* sp. (unresolved)	heart muscle: meronts (*L*)—diffuse
	ZA21/018	*H.* spp., *L.* spp. (unresolved co-infection)	heart muscle: meronts (*L*)—diffuse
*S. uralensis*	ZA21/071	*H. syrnii* hSTAL2	skeletal muscle: megalomeronts (*H*)—multifocal
	ZA21/105	*L.* sp. lSTAL7	kidney: meronts (*L*)—oligofocal

^a^ Reactivity of meronts with ISH probe(s) is given in parentheses (*H*: Haemo18S, specific to the genus *Haemoproteus*, *L*: Leuco18S, specific to the genus *Leucocytozoon*, *S*: STAL5-18S, specific to the *Leucocytozoon* lineage lSTAL5). ^b^ focal: meront(s) localized in a single focus; oligofocal: meronts found in one to three foci; multifocal: multiple meronts localized in more than three foci; diffuse: numerous meronts found widely spread.

## Data Availability

All generated sequence data were deposited in the NCBI GenBank and the MalAvi database. Any other data are available from the authors upon reasonable request.

## Supplementary Materials

The following supporting information can be downloaded at: https://www.mdpi.com/article/10.3390/ani12172212/s1, Figure S1: Reactive gliosis associated with a *Leucocytozoon* sp. lineage lSTAL5 meront cluster found in the brain of a Ural owl (*Strix aluco*).
