# Supplementary material for: Exo-Erythrocytic Development of Avian Haemosporidian Parasites in European Owls
Source: Animals (Basel). 2022 Aug 28;12(17):2212. doi: 10.3390/ani12172212 (PMC9454416; doi:10.3390/ani12172212)
Supplement: Supplementary file 1 [file animals-12-02212-s001.zip › animals-1848026-supplementary.pdf]

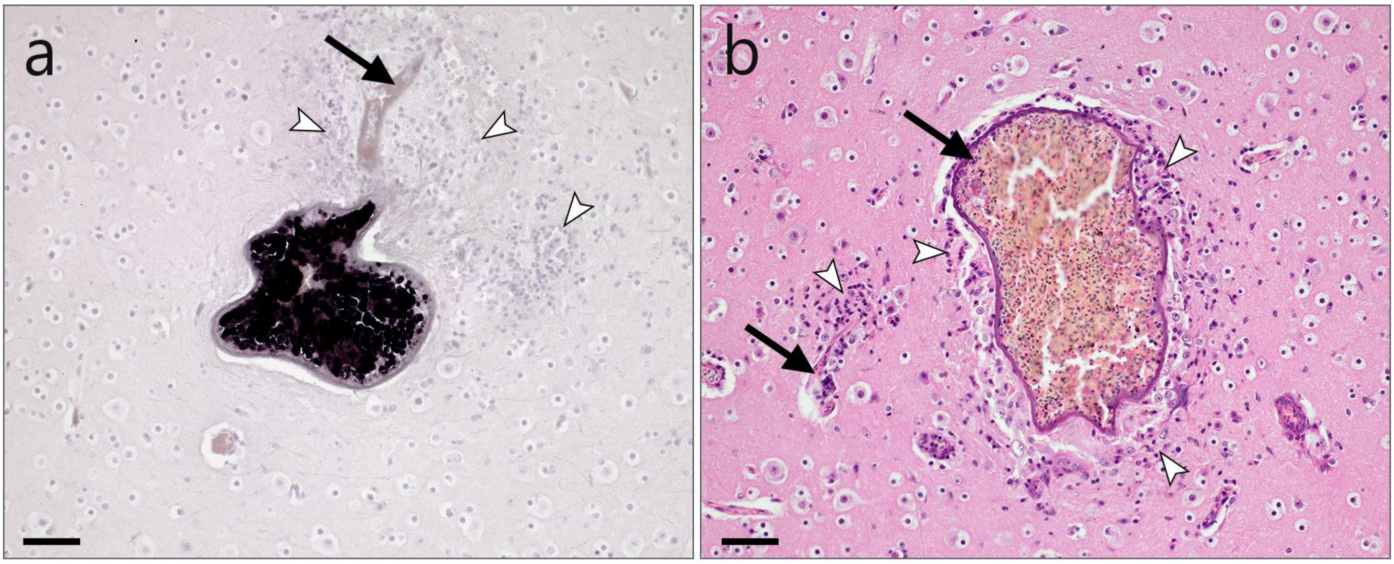

Figure S1. Reactive gliosis associated with a *Leucocytozoon* sp. (lineage ISTAL5) meront cluster in the brain of a Ural owl (*Strix aluco*). (**a**) A cluster of *Leucocytozoon* sp. (lineage ISTAL5) meronts was detected by chromogenic in situ hybridization using a ISTAL5 lineage-specific probe (dark purple staining). (**b**) Haematoxylin-eosin staining of an adjacent histological section depicting the same are as in (a), showing perivascular inflammation. Black arrow (➞)—blood vessel, white arrowhead (▷)—gliosis. Scale bars are 50um.
